# Globally Aging Cortical Spontaneous Activity Revealed by Multiple Metrics and Frequency Bands Using Resting-State Functional MRI

**DOI:** 10.3389/fnagi.2021.803436

**Published:** 2021-12-28

**Authors:** Xiu-Xia Xing

**Affiliations:** Department of Applied Mathematics, College of Mathematics, Faculty of Science, Beijing University of Technology, Beijing, China

**Keywords:** cortical spontaneous activity, aging, longitudinal design, amplitude, homogeneity, homotopy

## Abstract

Most existing aging studies using functional MRI (fMRI) are based on cross-sectional data but misinterpreted their findings (i.e., age-related differences) as longitudinal outcomes (i.e., aging-related changes). To delineate aging-related changes the of human cerebral cortex, we employed the resting-state fMRI (rsfMRI) data from 24 healthy elders in the PREVENT-AD cohort, obtaining five longitudinal scans per subject. Cortical spontaneous activity is measured globally with three rsfMRI metrics including its amplitude, homogeneity, and homotopy at three different frequency bands (slow-5: 0.02–0.03 Hz, slow-4: 0.03–0.08 Hz, and slow-3 band: 0.08–0.22 Hz). General additive mixed models revealed a universal pattern of the aging-related changes for the global cortical spontaneous activity, indicating increases of these rsfMRI metrics during aging. This aging pattern follows specific frequency and spatial profiles where higher slow bands show more non-linear curves and the amplitude exhibits more extensive and significant aging-related changes than the connectivity. These findings provide strong evidence that cortical spontaneous activity is aging globally, inspiring its clinical utility as neuroimaging markers for neruodegeneration disorders.

## 1. Introduction

MRI has advanced brain aging research and revealed consistent aging patterns of thinning cortical thickness and shrinking surface area (Elliott, 2020). These morphological changes are associated with cognitive development during the aging process (Cox et al., 2021). Meanwhile, in theory, only longitudinal design can delineate the aging-related changes. Recent study has demonstrated that cross-sectional design failed in reconstructing the aging-related changes but recovering the inter-individual differences in brain and mind (Vidal-Pineiro et al., 2021), i.e., the age-related differences. In the present study, the term “aging-related changes” refers to the longitudinal changes within individual subjects during the aging process while “age-related differences” refers to the cross-sectional changes mixing within- and between-subject differences. Therefore, cross-sectional studies do not only measure aging-related changes but have additional noise due to inter-individual differences. Longitudinal studies assess intra-individual changes, thus, getting rid of the latter problem (i.e., the mixing of inter-individual and aging differences). It highlights the need for longitudinal samples in building aging curves of human brain structure and function, i.e., modeling aging-related changes as a function of age.

Human brain function can be mapped with fMRI during either performing a specific task (i.e., common task-based fMRI, tbfMRI) or resting-state (rsfMRI). Both tbfMRI and rsfMRI can be highly or poorly reliable in characterizing individual differences in brain function (Noble et al., 2019; Elliott et al., 2020), depending on the metrics of use (Zuo and Xing, 2014; Kragel et al., 2021; Soch et al., 2021). TbfMRI is much better to detect aging or age-related changes associated with a specific cognitive function, although faces significant challenges of developing a single task valid for all different stages of aging, but it is, of course, less suited as a global measure for functional changes. In this regard, rsfMRI has no need for a complex experimental setting as tbfMRI and, thus, is more applicable across different aging stages (Biswal, 2012; Nooner et al., 2012) and more suitable for clinical conditions such as dementia or stroke (Raichle, 2006). Aging has been demonstrated to have significant effects on spontaneous brain activity measured by rsfMRI although the direction of these effects (i.e., increase vs. decrease) remains inconsistent among the studies (Ferreira and Busatto, 2013; Foo et al., 2020). This might be an indication of the limited reliability of the most common functional connectivity metrics (Noble et al., 2019) and the lack of longitudinal rsfMRI dataset (Zuo et al., 2014) in previous studies. A longitudinal work with reliable rsfMRI measurements is warranted for the insightful understanding of the aging spontaneous brain activity.

Neural oscillations occur across the whole cortical space and time according to a theoretical framework, namely the natural logarithm linear law (Penttonen and Buzsáki, 2003), parcellating them into multiple frequency bands of distinct physiological functions (Buzsaki and Draguhn, 2004). EEG and MEG offer great feasibility of recording these oscillations at high frequencies but with limited spatial resolution. In contrast, rsfMRI represents a high-spatial-resolution method to record the low-frequency (<0.1 Hz) oscillations of the blood oxygen level dependent (BOLD), which has been considered as the proxy of cortical spontaneous activity (CSA) (Fox and Raichle, 2007; Power et al., 2014). While early rsfMRI studies focused on a single frequency (e.g., 0.01–0.1 Hz) (Biswal, 2012), Zuo et al. were the first to decompose the rsfMRI signals into multiple frequency intervals according to the neural oscillation law (Zuo et al., 2010a). They demonstrated the specificity of the frequency band to human basal ganglia's spontaneous activity by directly comparing the rsfMRI amplitudes between two slow bands. Soon after, this multi-band method was used to untangle the spontaneous activity in mild cognitive impairments from normal aging (Han et al., 2011).

In the present study, the longitudinal rsfMRI data from the PREVENT-AD (PResymptomatic EValuation of Experimental or Novel Treatments for Alzheimer's disease) cohort (Tremblay-Mercier et al., 2021) were employed to build aging curves (i.e., aging-related changes) of global CSA at different spatial scales and across frequency bands. Specifically, three metrics are calculated for quantifying the CSA's amplitude of low frequency fluctuations (ALFF) (Zuo et al., 2010a), regional homogeneity (ReHo) (Zuo et al., 2013) and voxel-mirrored homotopic connectivity (VMHC) (Zuo et al., 2010b) across different slow bands (Xing et al., 2021). The author's tenet to use the global metrics was the simplicity, high test-retest reliability (Zuo and Xing, 2014), and potential validity (i.e., the observed age-related changes in previous study) (Zuo et al., 2010b; Wang et al., 2014; Jiang and Zuo, 2016; Zhao et al., 2020). The aging curves (i.e., derived with longitudinal data) remain elusive for the global CSA. According to the theory of neuronal oscillation (Buzsáki, 2009), different frequency bands are responsible for different types of connections and different levels of computation: slow oscillators favor long connections (low-level computation) while fast oscillators facilitate local integration (high-level computation). Therefore, combining the previous findings, the author hypothesized that the global CSA will show a universal aging pattern while (relatively speaking) higher frequency bands have more complex aging curves and (relatively speaking) more local metrics age more extensively across the frequency bands.

## 2. Materials and Methods

### 2.1. Participants and MRI Data

Twenty-four cognitively unimpaired older individuals with a parental or multiple-sibling history of AD were selected from the PREVENT-AD cohort (age range: 58–77 yrs; mean age: 66 yrs; 8 men). More details of these participants' demographics can be found in Supplementary Table 1 including years of education and APOE-ϵ4. While cognitive measures such as the Mini-Mental State Examination (MMSE) are not the focus of the current study, they are accessible from the PREVENT-AD (https://openpreventad.loris.ca). These participants are all from the observational cohort in PREVENT-AD and each received five longitudinal observations including the first annual visit (baseline) and follow-up visits at the 12, 24, 36, 48 months after the baseline visit. A final list of all the participants is available (subject ID: 1635604, 1776737, 2599481, 2823276, 2963960, 3165520, 3301724, 4040157, 4052945, 4943065, 5187625, 5692079, 5730499, 5979345, 7237992, 7550757, 7755697, 7760229, 7863867, 8120729, 8478383, 9088372, 9827494, 9909448). At each visit, all participants received a high-resolution (1 mm isotropic voxels) structural imaging scan with T1-weighted MP-RAGE sequence (TR = 2,300 ms, TE = 2.98 ms, TI = 900 ms, FA = 9°, FoV = 256 × 240 × 176 mm, Phase Encode: A-P, BW = 240 Hz/px, GRAPPA 2) and two 5-min identical-setting rsfMRI scans (4 mm isotropic voxels) with EPI sequences (TR = 2,000 ms, TE = 30 ms, FA = 90°, FoV = 256 × 256 mm, Number of Slices: 32, Eyes Status: closed).

### 2.2. Data Preprocessing

All preprocessing steps for both structural and fMRI images were implemented using the Connectome Computation System (CCS accessible at https://github.com/zuoxinian/CCS). This pipeline integrates multiple analytical software packages to achieve imaging processing of multi-modal MRI data with MATLAB implementations of three computational modules: data cleaning and preprocessing, individual connectome mapping and connectome mining, and knowledge discovery. The details of CCS can be found in its seminal software publication (Xu et al., 2015; Xing et al., in press). The present study only describes the image processing steps used. The structural image went through the following preprocessing steps: (1) spatially adaptive non-local means denoising, (2) rough inhomogeneity correction, (3) spatial normalization into the MNI standard brain space, (4) inhomogeneity correction, (5) intensity normalization, (6) brain extraction by non-local intracranial cavity extraction (NICE), and (7) gray and white matter segmentation, surface reconstruction. RsfMRI image preprocessing included (1) dropping off the first 5 EPI volumes, (2) removing and interpolating temporal spikes (i.e., despike), (3) correcting acquisition timing among image slices and head motion among image volumes, (4) normalizing the 4D global mean intensity to 10,000, (5) regressing out head motion artifacts and other spurious noise by using ICA-AROMA (Pruim et al., 2015a,b), and (6) removing linear and quadratic trends from the rsfMRI signals to mitigate the scanner-related (e.g., magnet instability or thermal noise) influences.

In the present study, the author constrains the data analyses to the cerebral cortex considering the advantages of surface-based functional brain mapping (Zuo et al., 2013; Coalson et al., 2018). All the structural and functional data were converted into the CIFTI-based grayordinate framework (Dickie et al., 2019). Spatial smoothing was not performed regarding the global nature of the present CSA analyses. Specifically, the preprocessed rsfMRI data are converted onto a left-right symmetric cortical surface grid, namely Conte69_LR32k. This surface template was reconstructed with the HCP-customized FreeSurfer pipeline based upon 69 healthy adults and comprises 32,492 vertices per hemisphere with an approximate 2 mm inter-vertex distance (Glasser et al., 2016). A group-level surface mask was established by including every vertex showing rfMRI signals from all the two rsfMRI scans of the 24 participants across the cortex.

### 2.3. CSA Metric Computation

To characterize the global CSA in the human brain across different spatial scales, the author calculated three widely used metrics: ALFF (Zang et al., 2007), ReHo (Zang et al., 2004), and VMHC (Zuo et al., 2010b). These metrics have been defined and described in detail in the recent study on test-retest reliability evaluation (Zuo and Xing, 2014; Chen et al., 2015). Specifically, the three metrics were calculated for three frequency bands: slow-5 band (0.02–0.03 Hz), slow-4 band (0.03–0.08 Hz), and slow-3 band (0.08–0.22 Hz), derived by the DREAM module in CCS (Buzsaki and Draguhn, 2004; Gong et al., 2021).

The CSA's ALFF at a single vertex was derived (~ within 2 mm distance) (Zuo et al., 2010a). The CSA's ReHo characterizes the local functional connectivity across the cortical mantle (Zuo et al., 2013). To quantify the ReHo vertex-wise, Kendall's coefficients of concordance (KCCs) of the rfMRI time series among the 4-step neighboring (~ 61 vertices within 16 mm distance) vertices were calculated. The CSA's VMHC was derived as the temporal correlation (Fisher-z transformed) between the rsfMRI timeseries from a pair of symmetric vertices between the two hemispheres (> 16 mm distance) (Zuo et al., 2010b). The author notes that these metrics measure CSAs across different spatial scales (i.e., from regional, local activity to distributional, distant connectivity) while they are also different in computational aspects: ALFF reflects the amplitude of the signals but ReHo and VMHC reflect correlations although all these are reflects of the underlying neural connections. For each rsfMRI scan, both the mean and SD of the raw maps for each metric are calculated within the group mask. These mean and SD values are then averaged across the two rsfMRI scans and considered as global CSA measurements at the three metrics and across the three frequency bands.

### 2.4. Aging Curve Modeling

For accurate estimation of the aging curves distinguishing longitudinal and cohort effects (Sørensen et al., 2021), generalized additive mixed models (GAMMs) include participant intercept as a random effect to model within-subject variability and sex, cohort, years of education as covariates (refer to Equation 1). Specifically, consider the current dataset of *n* = 24 participants indexed *i* = 1, ⋯ , *n*, assume an outcome of the global CSA *y*_*ij*_ has been measured *m*_*i*_ = 5 times in participant *i*, with timepoints indexed by *j* = 1, ⋯ , *m*_*i*_, and let *a*_*ij*_ denote the age of participant *i* at the *j*th timepoint.


(1) $$\begin{array}{r} y_{ij}^{\text{CSA}}=\beta_{0}+f\left(a_{ij}\right)+b_{0i}+\epsilon_{ij}, \end{array}$$

where *f*(*a*_*ij*_) models the effect of aging, β_0_ is the intercept, *b*_0*i*_ is the random intercept for participant *i*, and ϵ_*ij*_ is a random noise term. Both *b*_0*i*_ and ϵ_*ij*_ are assumed to be normally distributed but represented for between-participant (e.g., sex, education, and cohort) and within-participant residual variability. Specifically, the cohort is the decimal number of years between birth date and January 1, 1970. The *f*(*a*_*ij*_) is a smoothing function constructed with cubic B-splines (the knot number = 5). This setting was sufficient for adequate degrees of freedom across both spline terms from fittings of the model but also sufficient for reasonable computational efficiency. These GAMMs were used to characterize aging effects on the global CSA metrics with their aging curves. Given a CSA metric (ReHo, ALFF, VMHC) and a frequency band (slow-5, slow-4, slow-3), GAMMs can model it as a function of age using penalized smoothing splines with smoothing parameters, i.e., *f*(*a*), selected by restricted maximum likelihood. The GAMMs were implemented with **gamm** function from the *mgcv* package and relevant statistical reports were generated in R software (https://www.r-project.org).

## 3. Results

As hypothesized, the global CSA measurements exhibited universal aging effects across different metrics but distinct profiles at different frequency bands. Overall, no statistically significant effects of education were observed on the CSA measurements while cohort and sex demonstrated metric- and frequency-dependent effects on the global CSA measurements (refer to Supplementary Figures 1–4 for more details).

**Figure 1:**
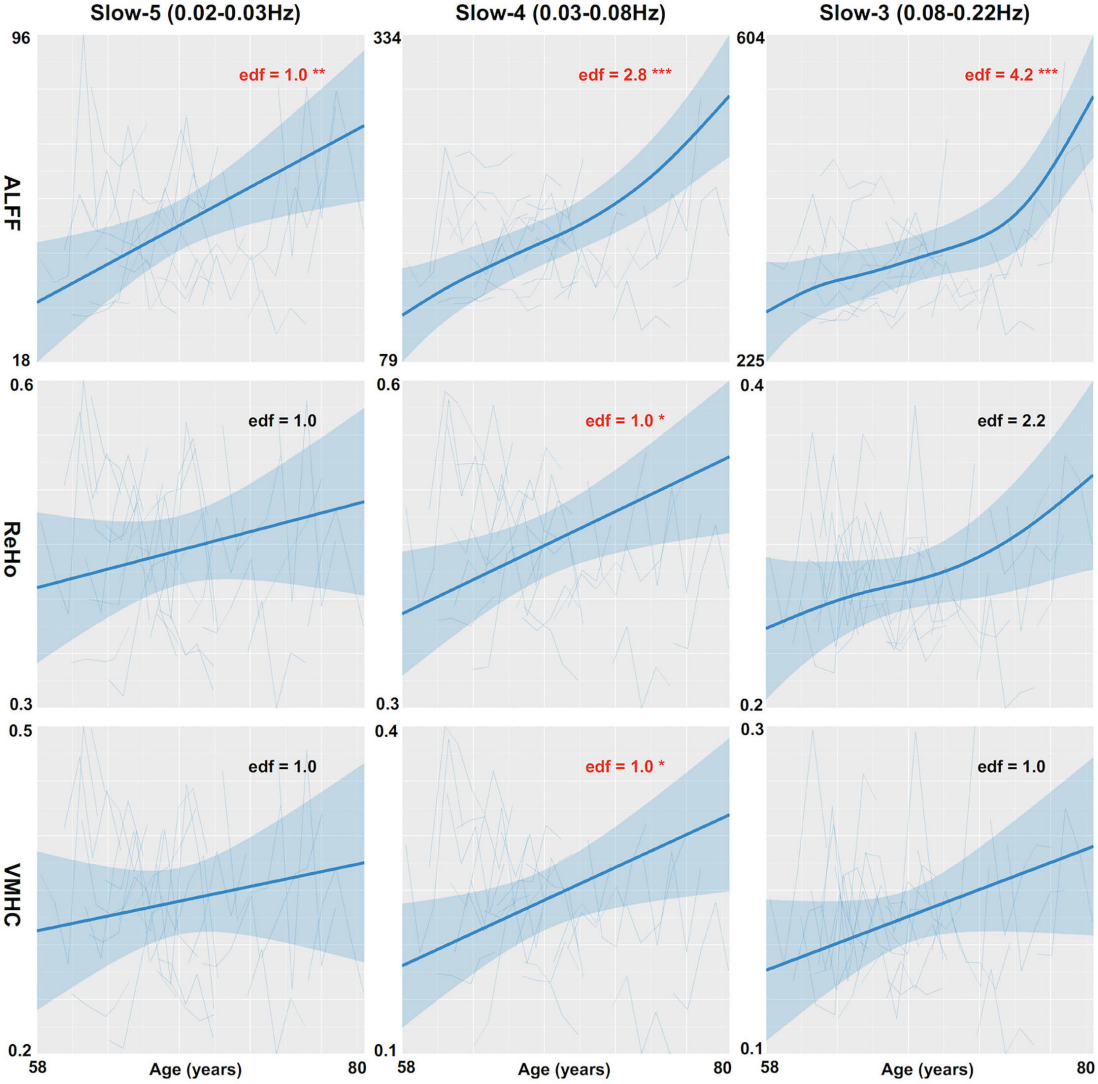
Global mean measurements of cortical spontaneous activity (CSA) are regressed against age. Three CSA metrics including ALFF (the top row), ReHo (the middle row), and VMHC (the bottom row) are plotted for each individual and modeled into the aging curves with the 95% CI (the shadow areas). The aging curve modeling is applied to three different frequency bands including slow-5 (the left column), slow-4 (the middle column), and slow-3 (the right column). edf, effective degree of freedom; *p < 0.05; **p < 0.01; ***p < 0.001.

**Figure 2:**
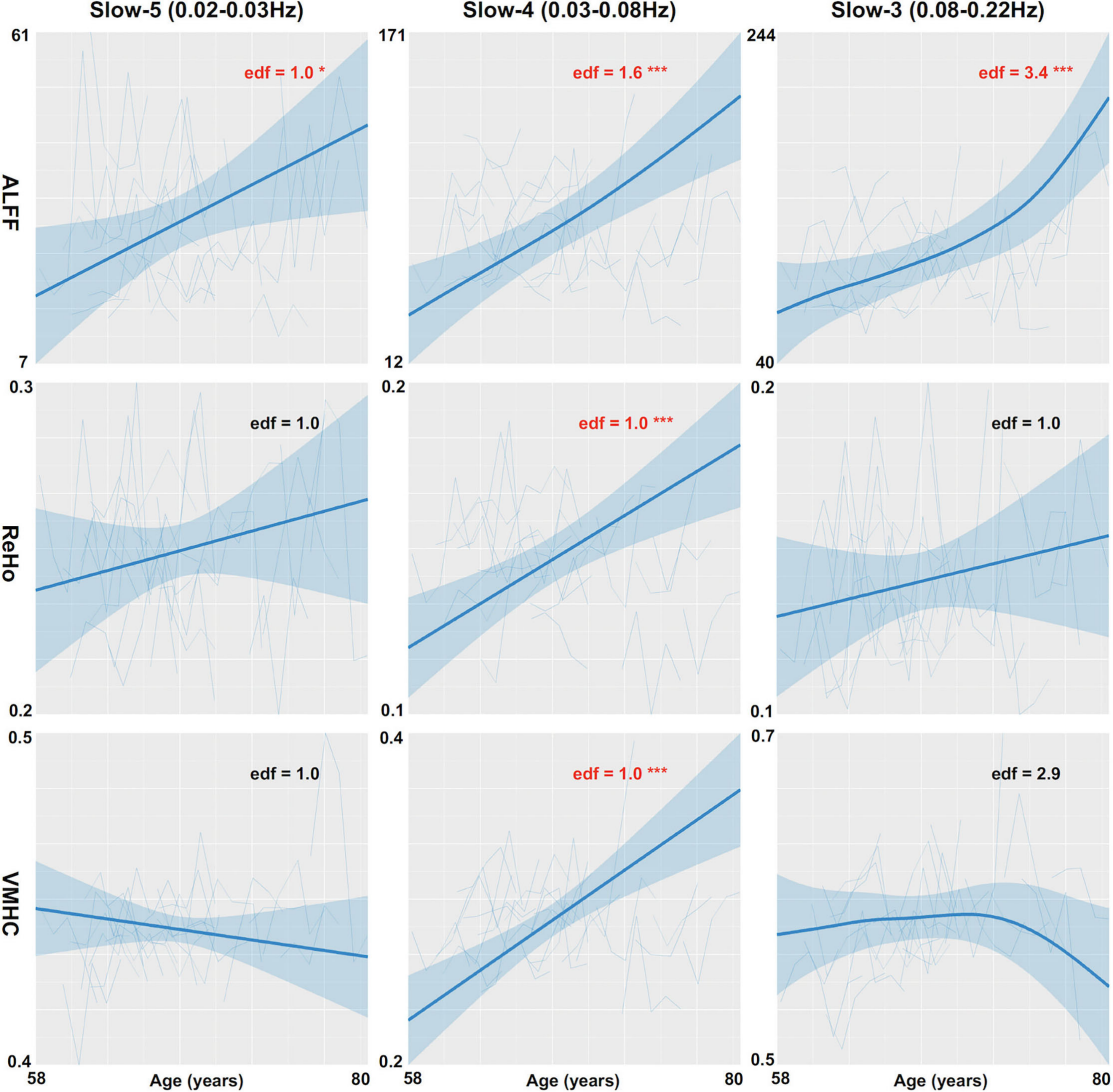
Global variability measurements of CSA are regressed against age. Three CSA metrics including ALFF (the top row), ReHo (the middle row), and VMHC (the bottom row) are plotted for each individual and modeled into the aging curves with the 95% CI (the shadow areas). The aging curve modeling are applied to three different frequency bands including slow-5 (the left column), slow-4 (the middle column), and slow-3 (the right column). edf: effective degree of freedom; *p < 0.05; **p < 0.01; ***p < 0.001.

The global mean ALFF measurements increased as a function of age (i.e., aging-related changes) for all the three frequency bands (the top panel in Figure 1, refer to full details of the statistical reports in Table 1). In slow-5, the aging curve was almost linear, which was indicated by the effective degree of freedom (edf) for describing the model complexity (*edf* = 1.0, *p* < 6.5 × 10^−3^). Younger cohorts were associated with higher ALFF (*p* < 0.013) and male participants showed higher ALFF than female participants (*p* < 0.011). In slow-4, the aging curve exhibited a weak two-stage non-linear profile where the aging velocity was higher in old participants (*edf* = 2.8, *p* < 5.6 × 10^−5^). Cohort (*p* < 2.1 × 10^−4^) and sex (*p* < 0.021) effects were similar to those in slow-5. In slow-3, the aging curve exhibited a strong two-stage non-linear profile where the aging velocity turned sharply at the age of 75 (*edf* = 4.2, *p* < 5.0 × 10^−5^) while cohort (*p* < 5.9 × 10^−3^) and sex (*p* < 9.6 × 10^−3^) had effects on ALFF in this frequency band. The global variability of ALFF measurements across the cortical mantle exhibited highly similar aging-related changes to the global mean ALFF (Figure 2, refer to Table 2 for details).

**Table 1:** Generalized additive mixed models (GAMMs) on aging curve models for global mean measurements of cortical spontaneous activity (CSA).

	**ALFF**			**ReHo**			**VMHC**		
Freq band	Slow-3	Slow-4	Slow-5	Slow-3	Slow-4	Slow-5	Slow-3	Slow-4	Slow-5
s(age).p	4.89E-05	5.63E-05	0.0064	0.15444	0.0130	0.2757	0.1093	0.0189	0.4222
s(age).edf	4.1988	2.8201	1.0000	2.20418	0.9999	0.9999	0.9999	1.0000	0.9999
Cohort.p	0.0058	0.0002	0.0127	0.05542	0.0027	0.0847	0.0823	0.0023	0.1269
Education.p	0.2734	0.5491	0.8797	0.712199	0.8512	0.9705	0.8950	0.8654	0.9412
Sex.p	0.0095	0.0200	0.0100	0.18635	0.3738	0.1252	0.1658	0.3825	0.1599

**Table 2:** Generalized additive mixed models (GAMMs) on aging curve models for global variability measurements of CSA.

	**ALFF**			**ReHo**			**VMHC**		
Freq band	Slow-3	Slow-4	Slow-5	Slow-3	Slow-4	Slow-5	Slow-3	Slow-4	Slow-5
s(age).p	0.0002	0.0001	0.0221	0.3570	0.0001	0.3204	0.1412	7.05E-06	0.3696
s(age).edf	3.4016	1.6285	0.9999	1.0000	1.0000	1.0000	2.9067	1.0000	1.0000
Cohort.p	0.0060	0.0002	0.0312	0.5336	0.0004	0.3208	0.1877	0.0002	0.0485
Education.p	0.4914	0.8217	0.7634	0.7454	0.6698	0.7575	0.8018	0.7700	0.6068
Sex.p	0.0331	0.0841	0.0139	0.2185	0.4688	0.2182	0.6023	0.2331	0.6635

The global mean ReHo and VMHC measurements increased as aging only for slow-4 (the middle and bottom panels in Figure 1 and Table 1). These aging curves were linear (*p*_ReHo_ <0.013, *p*_VMHC_ <0.019). These two connectivity metrics were not different between men and women but higher in younger cohorts ($p_{\text{ReHo}}<2.8\times 10^{-3},p_{\text{VMHC}}<2.4\times 10^{-3}$). The global variability of the two CSA connectivity measurements exhibited similar aging changes to their global means (refer to Table 2 for details). As an exception, the global VMHC variability of slow-5 was larger in younger cohorts (*p* < 0.05).

## 4. Discussions

In this report, the author observed a global aging pattern of CSA, which is universal for not only its amplitude but also its connectivity. These metrics share increases profiles during aging but exhibit specific frequency and spatial profiles. Higher frequency bands show more non-linear curves and the amplitude exhibits more extensive and significant aging-related changes than the connectivity. Age-related differences in CSA have been widely studied in older adults using cross-sectional design (Huang et al., 2015; Farras-Permanyer et al., 2019). The findings are diverse across spatial areas or networks and different age groups, leaving barries in their clinical translation. This may reflect the challenges of both the cross-sectional nature of the samples and the limited reliability of the functional connectivity metrics. The present study uses longitudinal design and delineates the aging-related changes of whole-brain CSA using highly reliable rfMRI metrics. It provides strong evidence that CSA is aging globally, which is not only inspiring for more insightful understandings of the healthy aging brain function but also sheds the light on its clinical utility for various neruodegeneration disorders by providing a normal reference.

A longitudinal design is a more powerful method for developing or aging research (Thompson et al., 2011), and has been considered as brain aging rather than the individual variations in brain age as revealed by cross-sectional design (Vidal-Pineiro et al., 2021). It has been rare for fMRI studies to have longitudinal data while structural or morphological MRI studies have reported the discrepancies of the findings between cross-sectional and longitudinal research (Ronnlund et al., 2005). In the present study, the author demonstrated the aging curves of the global CSA by taking the advantage of the open resource provided by the PREVENT-AD cohort. This seems to represent the first aging curve study using the richest longitudinal data (five repeated measurements) of the human brain function. As expected, the aging curves converged into a unified profile of increasing global amplitude, local and homotopic connectivity with age. The author speculates that it may indicate the underlying compensation mechanism on normal aging or neurodegeneration processes, calling for a confirmatory brain-mind association study in the future. It might also be novel for the current observation that the aging curves are dependent on space and frequency profiles, reflecting an aging rule of brain organization (Fox and Raichle, 2007; Zhang and Raichle, 2010).

Global metrics of the whole brain are commonly considered as measures of no interest, and, thus, overlooked for a long time, in most previous studies (e.g., Zhu et al., 2021) with very few exceptions (Zuo et al., 2010b). As the present findings support, the author argues that these global metrics are meaningful for modeling aging curves of the human brain function. The amplitude measure has been demonstrated with high test-retest reliability and validity of reflecting the organizational gradient and hierarchy of the human brain oscillations (Gong et al., 2021). Aging increases the CSA amplitude, and this may indicate the increasing need for energy to compensate for aging effects. Such aging-related increases are more complex and non-linear for the fast brain oscillations (e.g., slow-3 band), implying the higher-order cognitive function aged more severe in the late aging stage (Buzsaki and Draguhn, 2004; Raz and Rodrigue, 2006). The connectivity measures including both ReHo and VMHC also increase as aging but only in the slow-4 band. ReHo has been related to the functional hierarchy (i.e., segregation or integration of the information) (Jiang and Zuo, 2016) and the brain metabolism (Aiello et al., 2015; Bernier et al., 2017). The aging profiles of ReHo may, therefore, indicate degraded functional hierarchy of the intrinsic brain activity and its metabolic correspondence. From a perspective of functional homotopy, aging VMHC enhanced inter-hemispheric integration (cooperation) or weakened inter-hemispheric segregation (competition). The specificity of connectivity aging to slow-4 frequency band might be associated with the aging processes of some specific cognitive function such as working memory or language. This warrants future aging CSA-mind association studies. To summarize, these findings of the three global metrics converge into an aging model of the increasing amplitude and connectivity of the CSA.

Several limitations must be bared in mind while these global measures could be promising to serve individualized and personalized medicine. First, the sample size is relatively small while the longitudinal samples are substantial, and thus, a large group of participants is required to increase the generalizability of these GAMMs. Second, the participants are not fully healthy due to their family history of AD. This may cause the obvious cohort effects while enlarging the aging effects of interests. Third, only global features are examined in the present study, however, the observed aging curves should be interpreted with caution regarding their relationship with the more region-specific aging-related changes. This is because the regional aging-related changes could be highly variable across different spatial locations, leading to non-significant whole-brain aging curves. Meanwhile, mean curves of aging changes of metrics are not necessarily directly reflect the aging curves of mean metrics, especially for the non-linear aging-related changes. This deserves high-resolution vertex/region-wise, e.g., Shirer et al. (2012), CSA analyses of aging-related changes for future study.

## Data Availability Statement

The datasets analyzed for this study can be found in the PREVENT-AD open neuroscience platform (https://openpreventad.loris.ca).

## Ethics Statement

The studies involving human participants were reviewed and approved by PREVENT-AD Research Group, McGill University. The patients/participants provided their written informed consent to participate in this study.

## Author Contributions

The author confirms being the sole contributor of this work and has approved it for publication.

## Conflict of Interest

The author declares that the research was conducted in the absence of any commercial or financial relationships that could be construed as a potential conflict of interest.

## Publisher's Note

All claims expressed in this article are solely those of the authors and do not necessarily represent those of their affiliated organizations, or those of the publisher, the editors and the reviewers. Any product that may be evaluated in this article, or claim that may be made by its manufacturer, is not guaranteed or endorsed by the publisher.
