# Supplementary figures and images for: Globally Aging Cortical Spontaneous Activity Revealed by Multiple Metrics and Frequency Bands Using Resting-State Functional MRI
Source: Front Aging Neurosci. 2021 Dec 28;13:803436. doi: 10.3389/fnagi.2021.803436 (PMC8748263; doi:10.3389/fnagi.2021.803436)

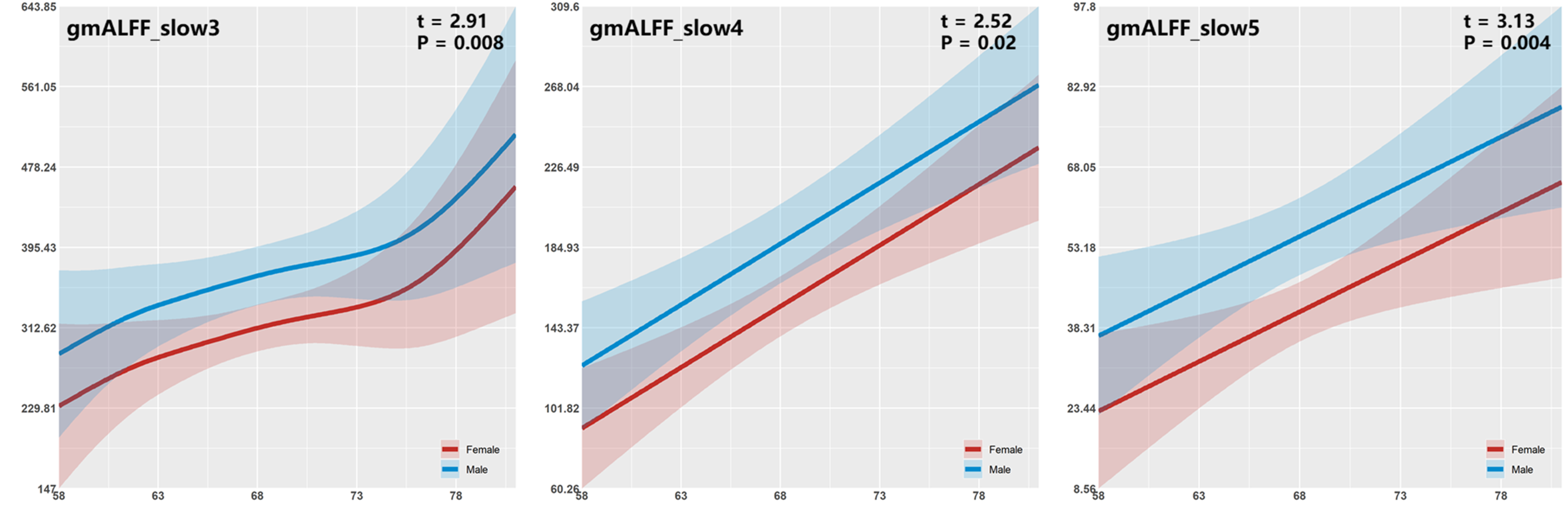

Supplement: Supplementary file 2 [file Image_1.PNG]

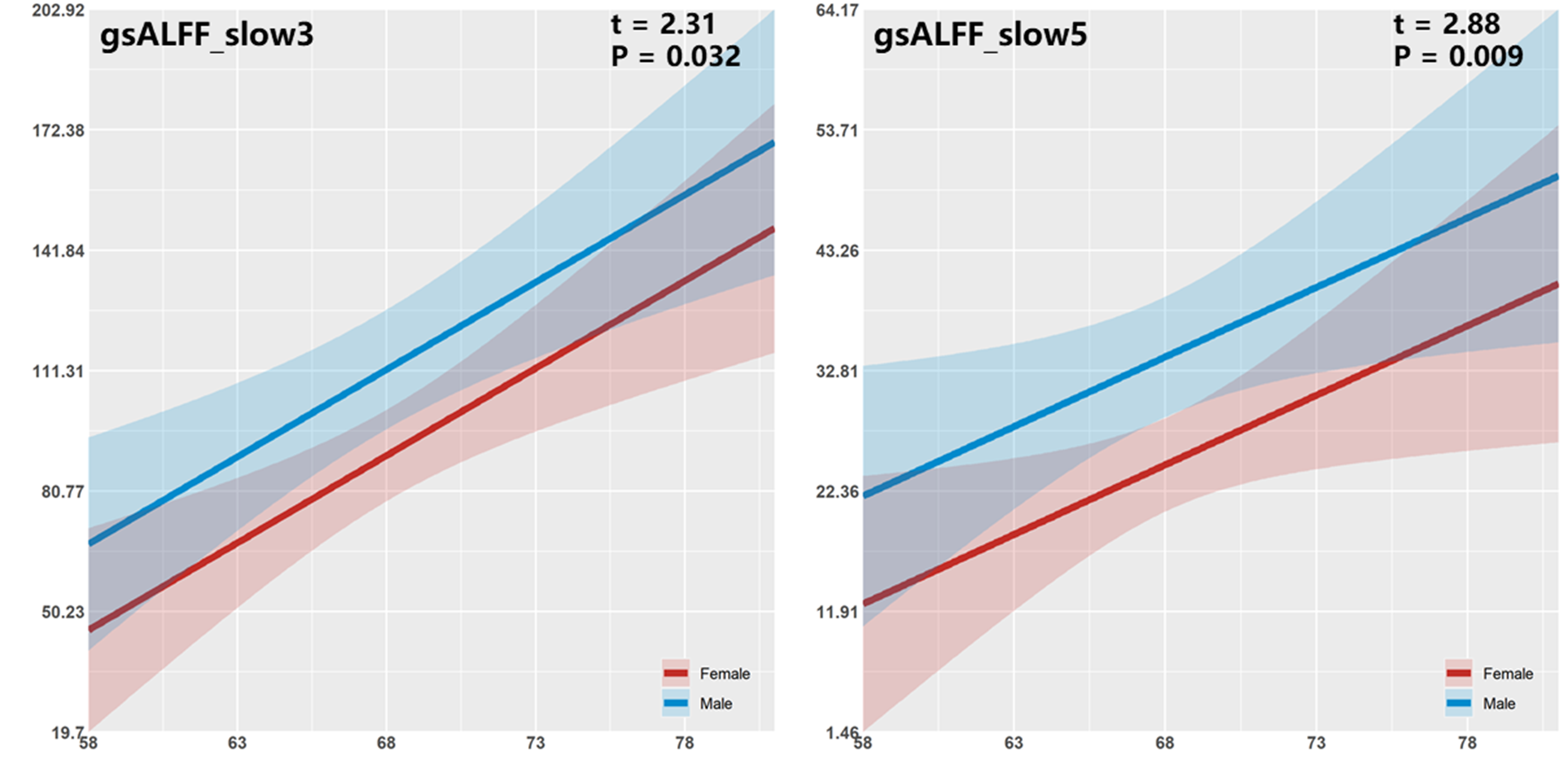

Supplement: Supplementary file 3 [file Image_2.PNG]

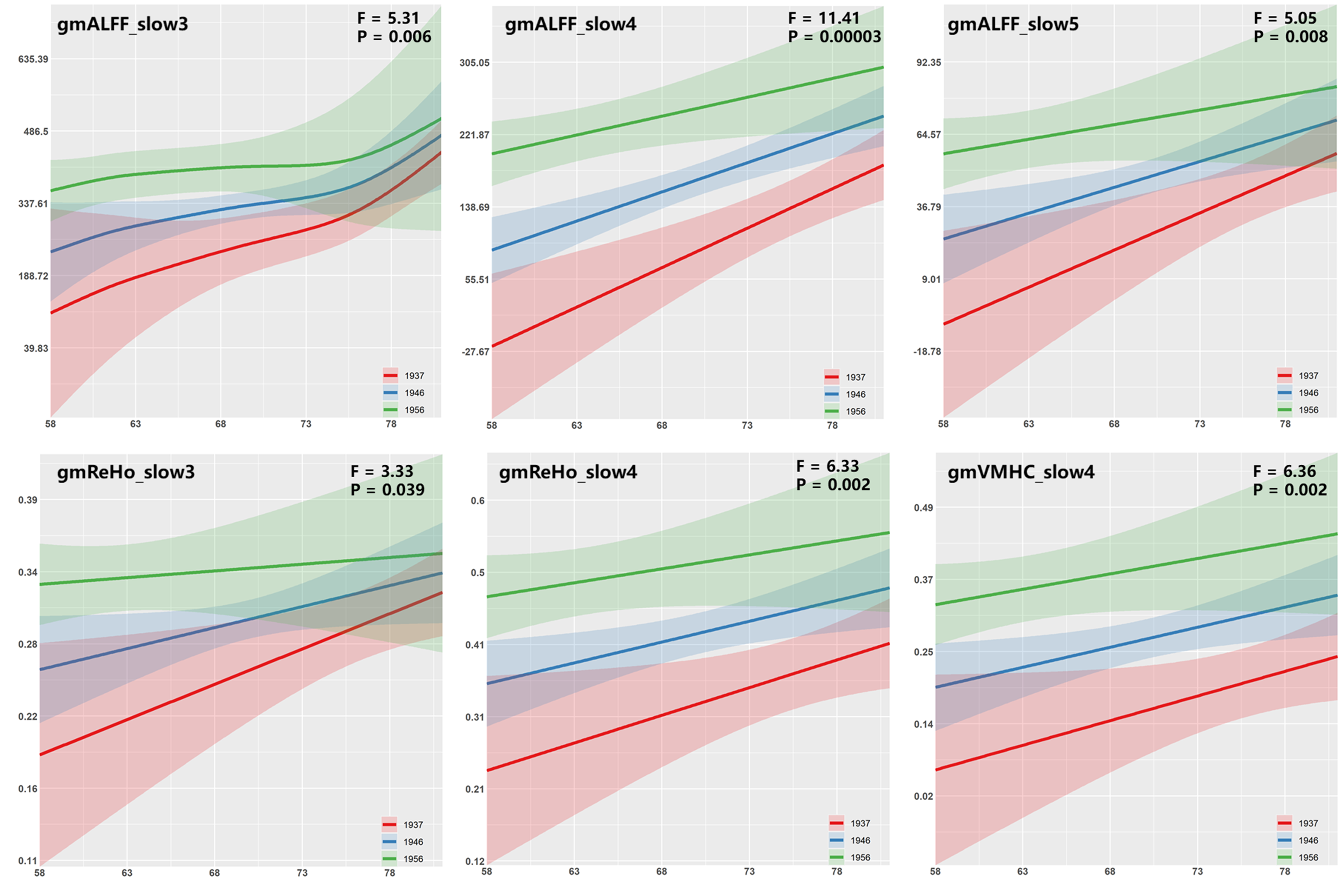

Supplement: Supplementary file 4 [file Image_3.PNG]

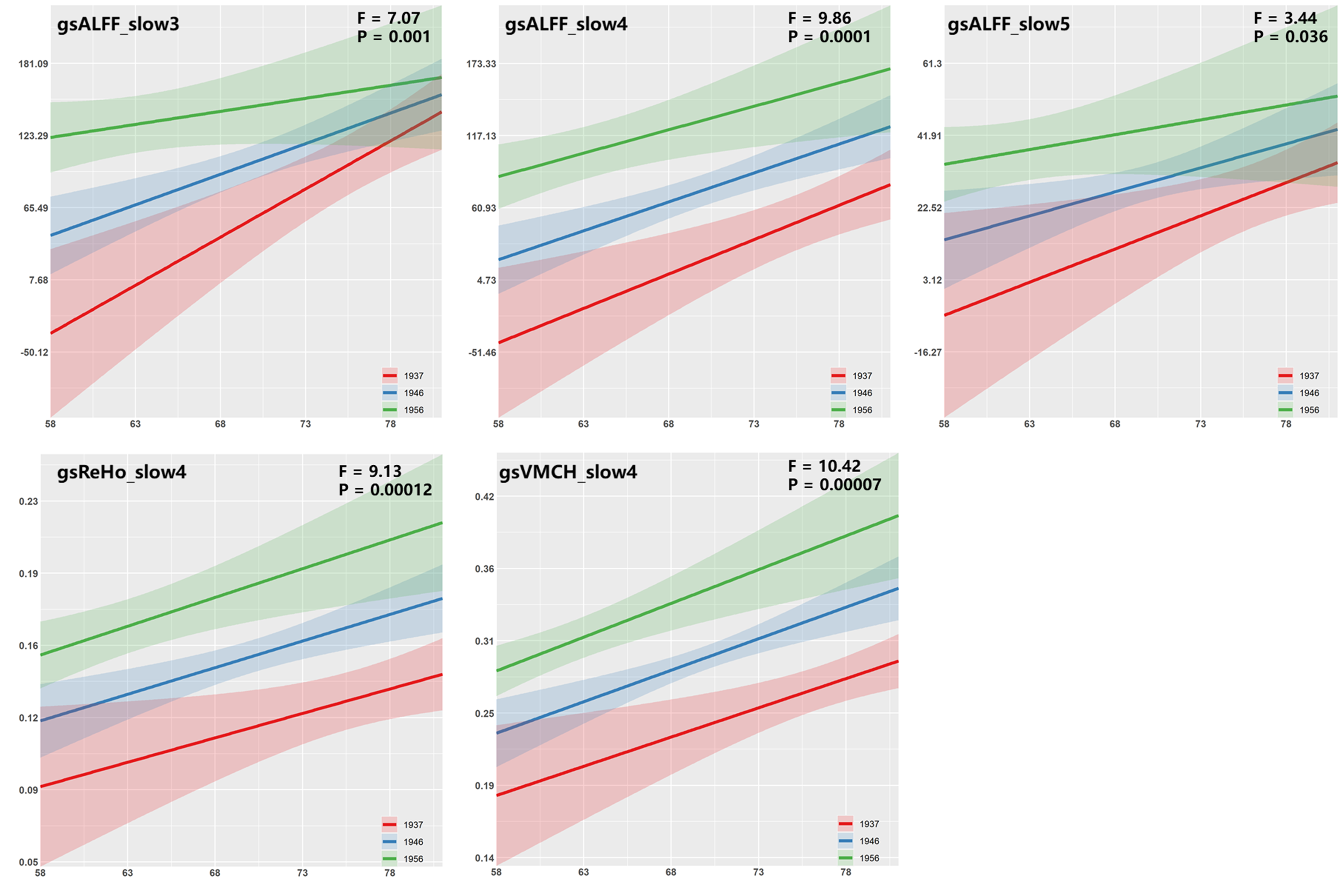

Supplement: Supplementary file 5 [file Image_4.PNG]
